# 1236. Implementation and Impact of a Pharmacist-driven Outpatient Antimicrobial Stewardship Program in a Costa Rican Private Hospital

**DOI:** 10.1093/ofid/ofad500.1076

**Published:** 2023-11-27

**Authors:** José P Díaz-Madriz, Esteban Zavaleta-Monestel, José M Chaverri-Fernández, Sebastián Arguedas-Chacón, Ariana Araya-Mena, Carolina Rojas-Chinchilla, Guillermo Fernández-Aguilar, Gabriel Muñoz-Gutierrez

**Affiliations:** Hospital Clínica Bíblica, San José, San Jose, Costa Rica; Pharmacy Director, Clínica Bíblica Hospital, San José, San Jose, Costa Rica; University of Costa Rica, San José, San Jose, Costa Rica; Pharmacy Intern, University of Costa Rica, San José, San Jose, Costa Rica; University of Costa Rica, San José, San Jose, Costa Rica; University of Costa Rica, San José, San Jose, Costa Rica; Hospital Clínica Bíblica, San José, San Jose, Costa Rica; Hospital Clínica Bíblica, San José, San Jose, Costa Rica

## Abstract

**Background:**

Antimicrobial stewardship programs (AMS) have been shown to be beneficial in both inpatient (IP) and outpatient (OP) settings. The CDC set the core elements for OP AMS initiation. In Latin America, progress has been made about AMS, but mainly in the IP. This hospital has an IP AMS that lacks activities dedicated to OP prescriptions. After conducting a baseline characterization of the OP antibiotics (ABs) prescriptions received in the hospital pharmacies, it was identified that ABs were prescribed mostly for urinary tract infections (UTIs).

**Methods:**

A retrospective observational analysis of the implementation of an OP AMS (Apr.-June 2022) and the impact of this program on the prescribing patterns for the management of OP UTIs. The level of adherence to CDC's core elements was assessed before and after the implementation. The impact in OP UTIs was estimated by comparing prescribing patterns according to the hospital clinical guideline in the emergency ward (ER) preAMS (July 2021-Mar. 2022) and postAMS (July 2022-Dec. 2022).

**Results:**

Compliance with CDC only met 28.6% of the core elements in the preAMS period, a percentage that improved to 85.7% after the implementation (Fig.1). For the impact on UTIs, 269 patient cases were analyzed (53.2% preAMS and 46.8% postAMS). The optimal selection of ABs in the preAMS period was 53.8% (n=77) and 95.2% (n=120) in the postAMS with an improvement of 41.4% (p< 0.001). Ciprofloxacin optimal selection had a 31.3% (p=0.028) increase, while levofloxacin exhibited a 60.0% (p=0,027) increment. Additionally, in the postAMS, the overall use of nitrofurantoin increased 3.6 times and the use of TMP-SMX and fluoroquinolones was reduced by 92.9% and 48.3%, respectively (Table 1). By diagnosis, there was a 56.0% (p< 0.001) improvement in the optimal treatment of uncomplicated cystitis and 9.6% (p=0.05) for complicated cystitis (Table 1).

The ER physicians were evaluated according to compliance with the clinical guidelines for UTIs. The mean score in the preAMS period was 46.2% and 90.0% in the postAMS (Fig.2).

**Figure 1.**
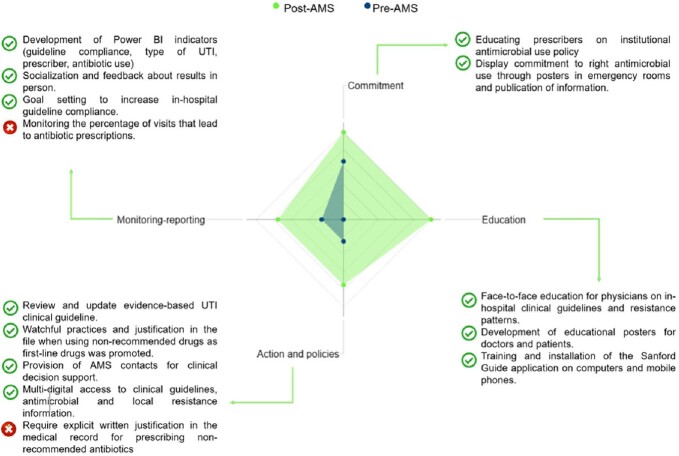
Compliance and activities related to the CDC Core Elements of outpatient AMS.

**Table 1.**
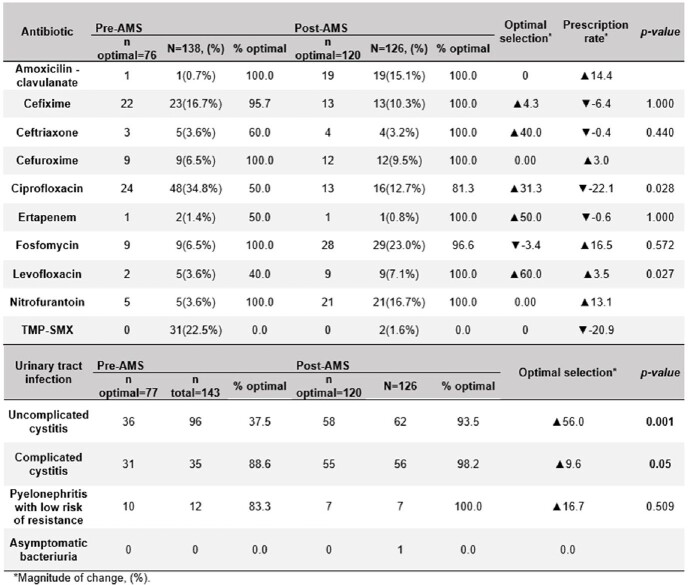
Comparison of optimal therapy selection in the emergency ward pre-AMS intervention and post-AMS by antibiotic prescription and UTI classification.

**Figure 2.**
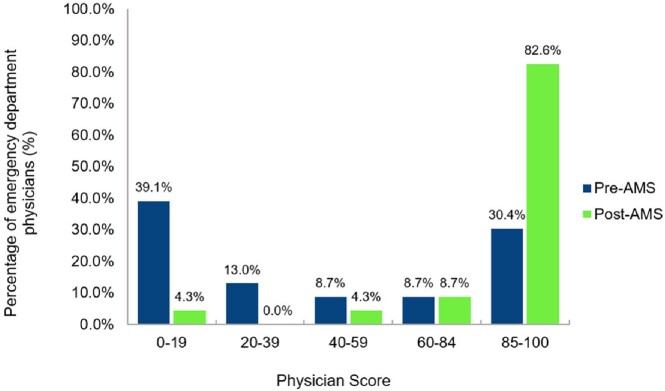
Distribution of scores of ER physicians according to adherence to clinical guidelines for UTI by period.

**Conclusion:**

The implementation of an OP-AMS according to CDC standards led to an improvement in complying with the OP UTI clinical guidelines. This suggests that this strategy could be applied to optimize the use of ABs in other infections in this population and region.

**Disclosures:**

**José P. Díaz-Madriz, PharmD**, Eli Lilly: Stocks/Bonds|MSD: Advisor/Consultant|Pfizer: Advisor/Consultant

